# Supplementary material for: Predicting the onset of internalizing disorders in early adolescence using deep learning optimized with AI
Source: Front Psychiatry. 2025 Oct 8;16:1487894. doi: 10.3389/fpsyt.2025.1487894 (PMC12547010; doi:10.3389/fpsyt.2025.1487894)
Supplement: Supplementary file 1 [file Supplementaryfile1.zip › Supplementary_Figures.docx]

Supplementary Material


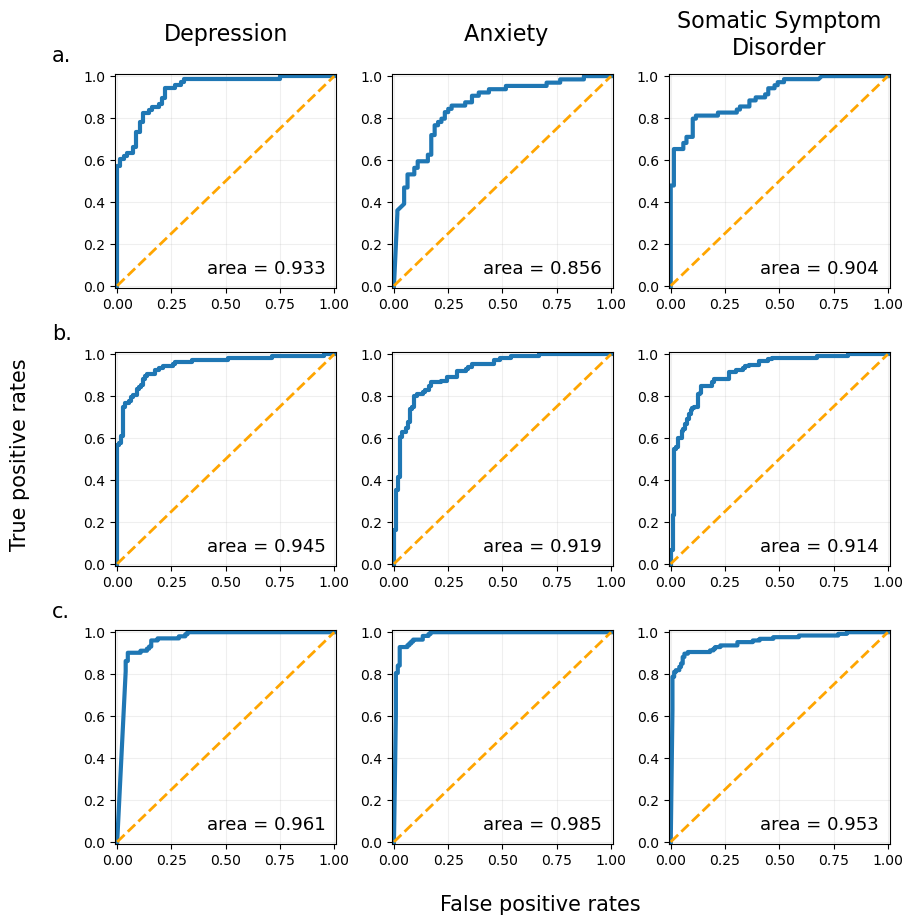


**Supplementary Figure 1.** Receiver operating characteristic curves (blue) are shown for a) new onset cases at 11-12 years of age; b) all prevailing cases at 11-12 years of age; and c) all prevailing cases at 9-10 years of age obtained with multimodal features input to deep learning optimized with Integrated Evolutionary Learning. The dashed lines (orange) represent random chance with an area of 0.5.


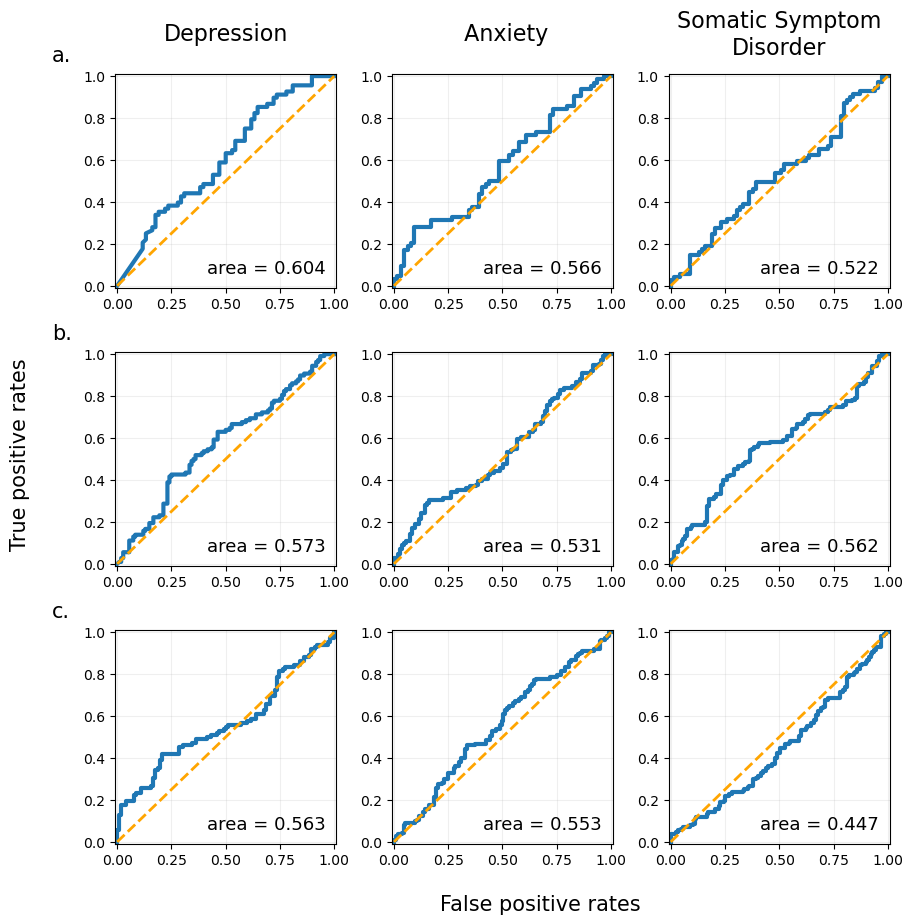


**Supplementary Figure 2.** Receiver operating characteristic curves are shown for a) new onset cases at 11-12 years of age; b) all prevailing cases at 11-12 years of age; and c) all prevailing cases at 9-10 years of age obtained with neural-only features input to deep learning optimized with Integrated Evolutionary Learning. The dashed lines (orange) represent random chance with an area of 0.5.
