# Supplementary figures and images for: Predicting the onset of internalizing disorders in early adolescence using deep learning optimized with AI
Source: Front Psychiatry. 2025 Oct 8;16:1487894. doi: 10.3389/fpsyt.2025.1487894 (PMC12547010; doi:10.3389/fpsyt.2025.1487894)

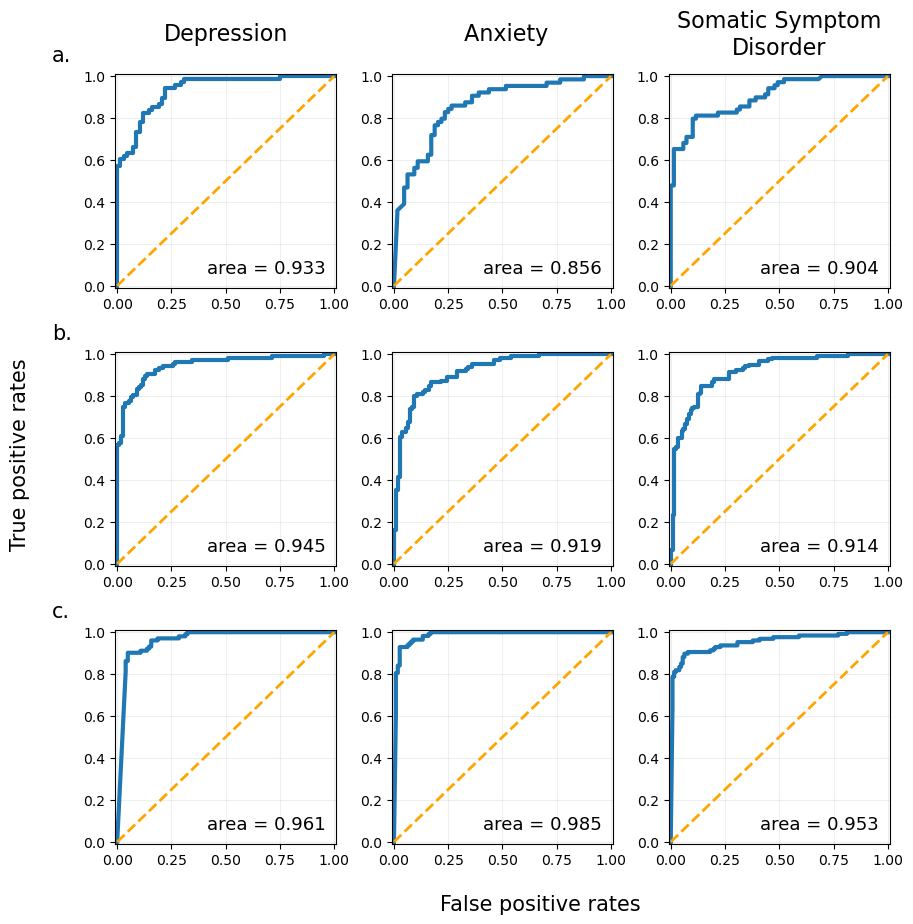

Supplement: Supplementary file 1 [file Supplementaryfile1.zip › Supplementary_Figure_1.tiff]

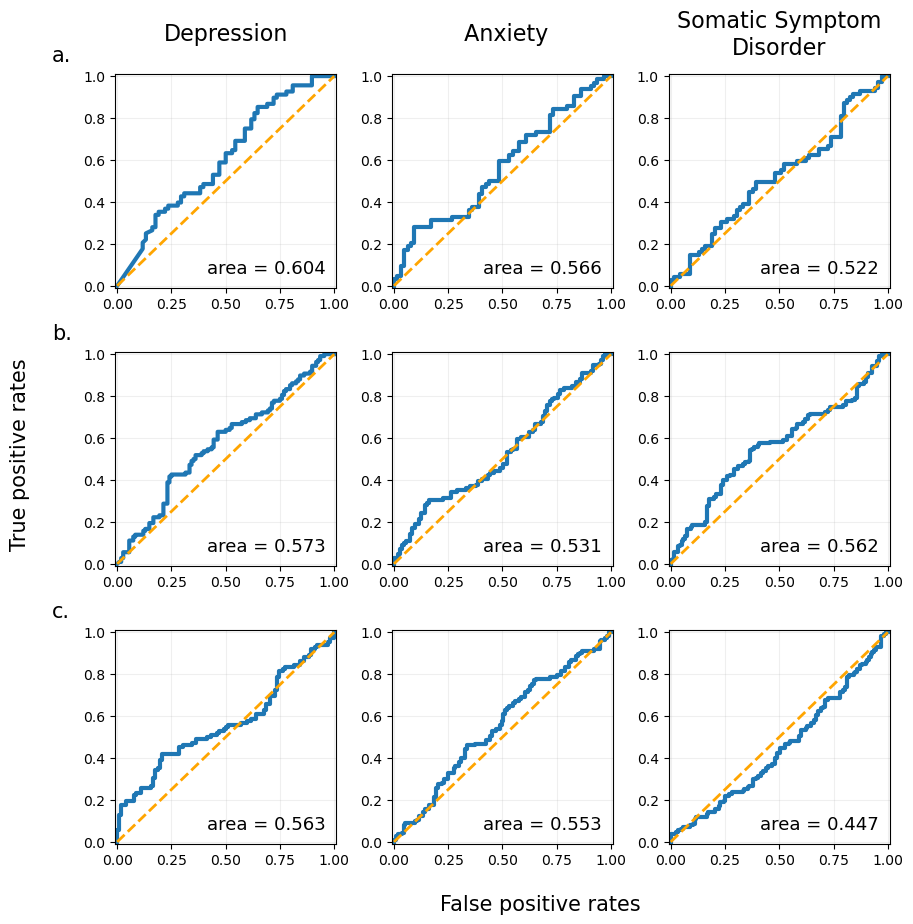

Supplement: Supplementary file 1 [file Supplementaryfile1.zip › Supplementary_Figure_2.tiff]
